# COVID-19 Pandemic: Demographic Predictors of Self-Isolation or Self-Quarantine and Impact of Isolation and Quarantine on Perceived Stress, Anxiety, and Depression

**DOI:** 10.3389/fpsyt.2021.553468

**Published:** 2021-02-01

**Authors:** Nnamdi Nkire, Kelly Mrklas, Marianne Hrabok, April Gusnowski, Wesley Vuong, Shireen Surood, Adam Abba-Aji, Liana Urichuk, Bo Cao, Andrew J. Greenshaw, Vincent I. O. Agyapong

**Affiliations:** ^1^Department of Psychiatry, Faculty of Medicine and Dentistry, University of Alberta, Edmonton, AB, Canada; ^2^Strategic Clinical Networks™, Provincial Clinical Excellence, Alberta Health Services, Calgary, AB, Canada; ^3^Department of Community Health Sciences, Cumming School of Medicine, University of Calgary, Calgary, AB, Canada; ^4^Cumming School of Medicine, University of Calgary, Calgary, AB, Canada; ^5^Addiction and Mental Health, Alberta Health Services, Edmonton, AB, Canada

**Keywords:** COVID-19, pandemic, stress, anxiety, depression, isolation, quarantine

## Abstract

**Introduction:** With the sudden onset and global dispersal of the SARS-CoV-2 virus, many nations including Canada attempted to reduce spread of the resultant COVID-19 syndrome with self-isolation and quarantine, while seeking a cure or vaccine for this disease. Understanding impacts of self-isolation and self-quarantine on stress, anxiety, and depression will help us to mitigate these issues through appropriate development of mental health services.

**Methods:** The sample was drawn from individuals who self-subscribed to Text4Hope, a service that delivers text messages based on a cognitive behavioral therapy framework. Text4Hope was developed to support Albertans during the COVID-19 pandemic. Subscribers were asked for demographic information and if they had to self-isolate or self-quarantine during the pandemic via a survey link. Mental health was assessed using the validated instruments: Perceived Stress Scale (PSS), Generalized Anxiety Disorder-7 item scale (GAD-7), and the Patient Health Questionnaire-9 (PHQ-9). Descriptive statistics and Chi-Square test results were derived using Statistical Package for Social Sciences (SPSS) version-26.

**Results:** 6,041 of 32,805 Text4Hope subscribers (18.4%) completed the survey. Of these respondents, 19.2% had self-isolated or self-quarantined in Alberta as of March 31, 2020 during the COVID-19 pandemic. *Post-hoc* analysis using adjusted residuals suggested that individuals aged 60 years of age or older, and retirees had a higher likelihood of self-isolation or self-quarantine, compared to respondents with other age or employment characteristics. One-week prevalence rates for self-reported measures of moderate to high stress, likely Generalized Anxiety Disorder (GAD), and likely Major Depressive Disorder (MDD) were 84.9, 46.7, and 41.4%, respectively. Respondents who had to self-isolate or self-quarantine during the COVID-19 pandemic were significantly more likely to present with moderate to high stress, significant anxiety symptomatology, and significant depressive symptomatology.

**Conclusions:** Older age and employment status were significantly associated with the likelihood of self-isolation or self-quarantine. We found elevated self-reported levels of anxiety and depression associated with self-reported COVID-19 pandemic-related self-isolation and self-quarantine activity. These findings have mental health implications both during and after the pandemic and demonstrate the need for greater focus on psychological complications of self-isolation and self-quarantine, and development of optimal ways to manage these pandemic consequences.

## Introduction

Coronavirus disease (COVID-19) is primarily a respiratory disease caused by severe acute respiratory syndrome coronavirus 2 (SARS-CoV-2) (1, 2). Its origin traces back to Wuhan, China where it was first reported in December 2019 (1, 2). Following initial reports, it rapidly spread globally and was declared a pandemic by the Word Health Organization (WHO) on March 11, 2020 (1). Nations soon took steps to reduce its spread by limiting access to certain facilities; implementing workplace hazard controls; introducing curfews and physical distancing measures, which include self-isolation and self-quarantine; imposing travel restrictions; and closing schools, shopping malls, and public spaces. These restrictions have caused substantial concerns, including the disruption of sport, recreation, and religious events, which individuals turn to for psychological and spiritual nourishment and entertainment. Panic buying was also observed, leading to a shortage of essential products, including toilet rolls and hand sanitizers.

Similar to other nations, Canada took steps to limit COVID-19 spread (2). Screening requirements were implemented for travelers returning from China to major airports in Montreal, Toronto, and Vancouver (January 22, 2020), later expanding to 10 airports in 6 provinces in early February 2020 (2). By mid-March 2020, voluntary, 2-week self-isolation was recommended for all individuals returning from travel abroad, a condition that was later mandated under the Canadian Federal Quarantine Act (March 25, 2020) (2). Self-isolation was also mandated for individuals diagnosed with COVID-19 and those who presented with disease-suggestive symptoms. Physical distancing measures, including maintenance of 2 m personal distance from others, and avoiding congregation, were put in place.

Self-isolation and self-quarantine measures deployed in the COVID-19 pandemic are based on public health knowledge gleaned from previous epidemics (3). Self-isolation, which is the separation of people who have been diagnosed with a contagious disease from those who are not sick (4, 5), differs from quarantine. Quarantine involves the separation and limitation of movement of individuals who have potentially been exposed to a contagious disease to see whether they become unwell, thereby reducing the risk of infection to others (3, 6). As well-intentioned and effective as these measures are, they produce unintended consequences. Previous research has shown that isolation and quarantine may increase suicidality (7). escalate boredom, heighten fears of infecting family, particularly among those with young children, limit supplies of essential goods, affect family finances, induce frustration, anger, and litigation (4, 7), and in some circumstances, result in the stigmatization of affected individuals.

Canada closed its southern border with the United States to all non-essential freight, services, and workers on March 18, 2020 (2). Similar mitigation strategies cascaded through Canadian provinces in order to limit COVID-19 spread. Provincial orders closed public schools, higher learning institutions, and daycare (March 15, 2020), followed by the closure of non-essential businesses (8). On March 25, 2020, Albertans were legally required under public health order to self-isolate after travel and when exhibiting symptoms, and to follow social distancing measures by limiting social interactions in public spaces, and maintaining 2 m personal distance from others (8).

Widespread public health efforts to try and limit rapidity of spread, to avoid overwhelming the healthcare system, caused cascading economic concerns as companies shut down and workers were laid off. Such economic worries are compounded by restrictions in social activities, limiting individuals to interaction primarily within their family units, including remote working for non-essential workers. Taha et al. showed that threats, such as those related to pandemics, affect physical health and cause psychological distress, with outcomes varying according to both appraisal and coping factors (3). They stress that emphasis on containment in family units and households gives rise to its own pressures, escalating pre-existing tensions in certain households, and/or giving rise to boredom. They also emphasize that individuals at home also may have increased time to tune in to media broadcasts, which are filled invariably with gloomy predictions about COVID-19 related morbidity and mortality and a degree of accompanying misinformation, both about the virus and disease spread. These factors produce fertile ground for mental illness, and anxiety may become exacerbated in ambiguous or uncertain situations (3).

Furthermore, ideas concerning treatment of COVID-19 remain ambiguous and varied, including considerable public debate evolving over use and types of face masks as a protective measure, with prevention based primarily on hand-washing and physical distancing. This creates palpable tension between fear for the present and uncertainty about the future. While the public health response is focused on the fight to reduce infection rates due to community transmission of COVID-19, the overriding clinical goal is to save lives. These twin approaches, coupled with uncertain outcomes, have exacted a toll on the mental health of patients, families, and healthcare workers, as well as the population at large. Pandemic measures adopted so far, while effective, may also have widespread mental health implications that have not yet been examined. Mental health status, in turn, is likely to have significant effects on how well the population is able to comply with self-isolation and self-quarantine requirements.

Text messaging has demonstrated efficacy in public health interventions (9). To help address the potential stressors and mental health difficulties which inevitably arise during emergencies, Alberta Health Services (AHS), in conjunction with six health foundations and the Department of Psychiatry at the University of Alberta, launched the Text4Hope program. Text4Hope is an evidence-based tool providing daily free, cognitive behavioral therapy-based text messages for 3 months to Albertans who subscribe. Launched in March 2020, Text4Hope evolved from the pre-existing program infrastructure supporting the Text4Mood program, initially launched in January 2016 (10), and deployed to support patients in the aftermath of the Fort McMurray wildfires in 2016. Text messages were written by mental health therapists and were further refined from patient feedback. They are intended to help individuals identify and adjust negative thoughts, feelings, and behaviors arising from the COVID-19 pandemic. Understanding the extent of self-isolation and self-quarantine on stress, anxiety, and depression can help decision makers plan and allocate mental health resources more effectively for this, and future pandemics. This study examined the demographic characteristics of Albertans who self-isolated or self-quarantined during the early stages of the COVID-19 pandemic, and assessed the influence of self-isolation or self-quarantine on stress levels, anxiety, and depression.

## Methods

We conducted a cross-sectional survey with online data collection. The study was approved by the University of Alberta Human Research Ethics Review Board (Pro00086163); consent to participate was implied when participants completed and submitted online survey responses. Demographic and clinical data were collected from subscribers to the Text4Hope program which was launched March 23, 2020 by the Chief Medical Officer for the Government of Alberta as part of the official government COVID-19 announcements. Using posters, email, and social media, individuals were invited to subscribe to the program by texting “COVID19HOPE” to a short-code number. Subscribers then received a survey link to assess respondent self-isolation or self-quarantine activity during the COVID-19 pandemic and gather demographic characteristics including age, gender, ethnicity, education, employment status, relationship status, and housing status. The survey assessed clinical correlates using validated tools: the Perceived Stress Scale (PSS, to measure stress with moderate to high stress indicated with scores 14 and higher) (11), the General Anxiety Disorder-7 item scale (GAD-7) (12), to measure anxiety with likely Generalized Anxiety Disorder (GAD) represented by scores of 10 and higher), and the Patient Health Questionnaire-9 scale (PHQ-9, to measure depression with likely Major Depressive Disorder (MDD) represented by scores 10 and higher) (13). An *a priori* sample size of 4,200 was required to estimate mental disorder prevalence rates in Alberta (2019 population: 4,371,316) with a confidence level of 99% and 2% margin of error. With an expected 20% response rate (10), we planned to extract data upon recruitment of at least 20,785 Text4Hope subscribers. Data were extracted 1-week post-program launch with a total of 32,805 active Text4Hope subscribers. We analyzed data with Statistical Package for Social Sciences (SPSS) version 26 (IBM 2019) to generate descriptive statistics and Chi-square tests. Two-tailed significance (*p* < 0.05) was used to assess the relationship between self-isolation or self-quarantine activity and clinical outcomes. There was no imputation for missing data and the results are based solely on completed survey responses.

## Results

Of the 32,805 individuals subscribed to Text4Hope at 1-week post-launch, 6,041 individuals (18.4%) completed the baseline online survey. Overall, 86.4% of respondents identified as female (*n* = 5,185) and 19.2% of respondents (*n* = 1,126) self-isolated or self-quarantined by March 31, 2020 as part of public health measures put in place to contain the spread of the coronavirus in Alberta during the COVID-19 pandemic. Table 1 provides descriptive summaries of respondent demographics and clinical correlates.

**Table 1:** Gender distribution of demographic and clinical characteristics of respondents.

**Variables**	**Male *N* (%)**	**Female *N* (%)**	**Gender Diverse *N* (%)**	**Overall *N* (%)**
**Age (Years)**				
≤25	74 (10.3)	550 (10.8)	15 (30.6)	639 (10.9)
26–40	247 (34.3)	1,905 (37.4)	21 (42.9)	2,173 (37.1)
41–60	308 (42.8)	2,213 (43.5)	11 (22.4)	2,532 (43.2)
>60	91 (12.6)	423 (8.3)	2 (4.1)	516 (8.8)
**Ethnicity**				
Caucasian	560 (75.8)	4,307 (83.5)	37 (61.7)	4,904 (82.3)
Indigenous	20 (2.7)	180 (3.5)	4 (6.7)	204 (3.4)
Asian	73 (9.9)	226 (4.4)	1 (1.7)	73 (5.0)
Other	86 (11.6)	448 (8.7)	18 (30.0)	86 (1.4)
**Education**				
Less than High School Diploma	43 (5.8)	166 (3.2)	8 (13.1)	217 (3.6)
High School Diploma	93 (12.6)	483 (9.3)	6 (9.8)	582 (0.6)
Post-Secondary Education	598 (81.0)	4,476 (86.5)	41 (67.2)	5,115 (85.6)
Other Education	4 (0.5)	49 (0.9)	6 (9.8)	59 (1.0)
**Employment status**				
Employed	509 (73.2)	3,188 (72.2)	24 (57.1)	3,721 (72.2)
Unemployed	89 (12.8)	618 (14.0)	9 (21.4)	716 (13.3)
Retired	59 (8.5)	336 (07.6)	1 (2.4)	396 (07.7)
Students	38 (5.5)	275 (06.2)	8 (19.0)	321 (06.2)
**Relationship status**				
Married/cohabiting/partnered	521 (70.7)	3,728 (72.1)	28 (45.9)	4,277 (71.6)
Separated/Divorced	37 (5.0)	400 (7.7)	1 (01.6)	438 (7.3)
Widowed	6 (0.8)	85 (1.6)	0 (00.0)	91 (1.5)
Single	167 (22.7)	916 (17.7)	21 (34.4)	1,104 (18.5)
Other	6 (0.8)	45 (0.9)	11 (18.0)	62 (01.0)
**Housing status**				
Own home	457 (63.6)	3,427 67.3)	23 (37.7)	3,907 (66.6)
Living with family	74 (10.3)	460 (9.0)	13 (21.3)	547 (09.3)
Renting	184 (25.6)	1,115 (22.6)	20 (32.8)	1,354 (23.1)
Other				
**Self-isolated/self-quarantined**	118 (16.5)	996 (19.6)	12 (20.7)	1,126 (19.2)
**Respondents reported moderate/high stress**	521 (79.2)	4,115 (81.6)	47 (90.5)	4,683 (84.9)
**Respondents reported significant GAD symptoms**	243 (40.9)	2,087 (47.3)	30 (62.5)	2,360 (46.7)
**Respondents reported significant depressive symptoms**	228 (37.3)	1,874 (41.8)	27 (56.3)	2,129 (41.4)

One-week prevalence rates for moderate to high stress, likely GAD, and likely MDD in Alberta were 84.9% (*n* = 4,683), 46.7% (*n* = 2,360), and 41.4% (*n* = 2,129), respectively.

Table 2 suggests there were significant associations between need to self-isolate and self-quarantine with two demographic variables: age and employment status. Specifically, individuals >60 years of age and retirees had a higher likelihood of self-isolation or self-quarantine, compared to respondents with other age or employment characteristics.

**Table 2:** Chi-Squared-test of association between demographic characteristics and likelihood to self-isolate or self-quarantine.

**Variables**	**Had to self-isolate or self-quarantine (*N* = 1,126)**	**Have not had to self-isolate or self-quarantine (*N* = 4,915)**	**Chi-Square**	***P*-value**
**Gender**				
Male	118 (16.5%)	595 (83.5%)	3.80	0.15
Female	996 (19.6%)	4,089 (80.4%)		
Gender diverse	2 (20.7%)	46 (79.3%)		
**Age (Years)**				
≤25	113 (20.9%)	496 (79.1%)	21.57	0.00
26–40	428 (20.0%)	1,710 (80.0%)		
41–60	424 (17.0%)	2,073 (83.0%)		
>60	126 (25.2%)	374 (74.8%)		
**Ethnicity**				
Caucasian	948 (19.7%)	3,871 (80.3%)	4.75	0.19
Indigenous	37 (18.7%)	161 (81.3%)		
Asian	52 (18.1%)	236 (81.9%)		
Other	85 (15.9%)	450 (81.4%)		
**Education**				
Less than High School Diploma	44 (21.0%)	116 (79.0%)	1.57	0.67
High School Diploma	101 (17.8%)	468 (82.2%)		
Post-Secondary Education	969 (19.3%)	4,053 (80.7%)		
Other Education	9 (16.1%)	47 (83.9%)		
**Employment status**				
Employed	645 (17.6%)	3,014 (82.4%)	18.44	0.00
Unemployed	151 (21.4%)	553 (78.6%)		
Retired	98 (25.7%)	283 (74.3%)		
Students	60 (18.9%)	257 (81.1%)		
**Relationship status**				
Married/cohabiting/partnered	801 (19.1%)	3,398 (80.9%)	0.71	0.95
Separated/Divorced	87 (20.1%)	346 (79.9%)		
Widowed	15 (16.7%)	75 (83.3%)		
Single	210 (19.5%)	866 (80.5%)		
Other	15 (16.7%)	75 (83.3%)		
**Housing status**				
Own home	721 (18.6%)	3161 (81.4%)	9.64	0.07
Living with family	95 (17.6%)	445 (82.4%)		
Renting	286 (21.6%)	1,058 (78.8%)		
Other	15 (25.0%)	45 (75.0%)		

Table 3 shows that respondents who had had to self-isolate or self-quarantine during the COVID-19 pandemic were significantly more likely to present with moderate to high stress, significant anxiety symptomatology, and significant depressive symptomatology. Small effect sizes were observed for each association.

**Table 3:** Chi-Square-test of association between self-isolation/self-quarantine and perceived stress, likely GAD, and likely MDD.

**Variables**	**Perceived Stress (PSS scale)**			**Generalized anxiety disorder symptoms (GAD-7 scale)**			**Major depressive disorder symptoms (PHQ-9 scale)**		
	**Moderate/High Stress *n* (%)**	***P*-value**	**Effect size (Phi)**	**GAD likely *n* (%)**	***P*-value**	**Effect size (Phi)**	**MDD Likely *n* (%)**	***P*-value**	**Effect size (Phi)**
**Self-isolated/quarantined**									
No	3,755 (84.3%)	0.03	0.030	1,849 (45.3%)	0.00	0.058	1,665 (40.1%)	0.00	0.052
Yes	925 (87.0%)			511 (52.6%)			461 (46.5%)		

## Discussion

This is the first Canadian study to survey the impact of self-isolation or self-quarantine measures on self-reported stress, anxiety, and depression, during the COVID-19 pandemic. The study included a large sample size as well as validated self-report measures. The majority of respondents in this survey identified as Caucasian (*n* = 4,904, 82.3%), between the ages of 26–60 years (*n* = 4,705, 80.3%), with post-secondary school education (*n* = 5,115, 85.6%), married or cohabiting (*n* = 4,277, 71.6%), employed (*n* = 3,721, 72.2%), and living in their own home (*n* = 3,907, 66.6%). These figures suggest pre-existing socioeconomic stability within the sample, prior to onset of the COVID-19 pandemic, thereby potentially mitigating the effects of these variables on their experiences of mental health difficulties. The underrepresentation of those aged 60 years and above in this study is similar to what is observed in other studies (14) and may limit our inferences for this cohort. Social isolation and quarantine are effective methods of achieving prevention of the spread of infectious diseases, yet also limit individual rights (15), and are associated with negative impacts on mental health. Some studies demonstrate negative psychological effects that outlast the pandemic (16, 17). A recent review by Brooks et al. (4) examining the psychological impact of quarantine suggested that the psychological impact of quarantine is substantial, wide-ranging, and long lasting.

Respondents who self-isolated or self-quarantined during this early COVID-19 pandemic stage were significantly more likely to present with moderate to high stress, likely GAD, and likely MDD, with small effect sizes for each association. The rates of anxiety (46.7%, *n* = 2,360) and depression (41.4%, *n* = 2,129) reported by respondents were substantially higher than both anxiety (6.3%) and depression (17.2%) rates reported by Wang et al. (18) in this context in China. Sensitization of the Canadian cohort by the free flow of information about the disease and its complications, as well as the inability to curtail disease transmission may have shaped the differences noted, in comparison to the Chinese sample.

Our study also showed that older adults and those who were retired were more likely to self-isolate or self-quarantine. This could reflect an increased likelihood of recent travel abroad (e.g., snow-birds or winter vacations) or may reflect greater awareness or knowledge of the risks to the elderly from reported COVID-19 fatalities, bearing in mind the greater preponderance of medical comorbidities within this age group. This is a hypothesis that merits further exploration. Self-isolation, boredom, increased access to pessimistic information about the pandemic, occupational disruption and financial difficulties, and an increased number of deaths amongst older adults may collectively pose a higher burden on those in this age cohort, thereby increasing their risk of stress, anxiety, and depression (19).

There are many documented socio-demographic factors that predict which individuals are more likely to be vulnerable to disaster-related mental health impacts, including lower individual resilience, poor coping skills, disaster severity, degree of victim involvement, pre-existing mental health issues, gender, age, social support and relationships, government and insurance company support, and duration of the mental health issue (20, 21). Our present study did not examine the effects of employment type on the likelihood for individuals to self-isolation or self-quarantine, or on the mental health effects of the pandemic. However, Qiu et al. (19) found that migrant workers experienced higher distress levels in China during the COVID-19 pandemic. Previous studies also reported that age likely increases the risk of development of mental illness following natural disasters (22) and in particular, that individuals between the ages 18–30 years and those aged above 60 years were at increased risk of distress during the COVID-19 pandemic (19). Although individuals that self-identified as female may exhibit substantially increased anxiety risk (three-fold greater than self-identified males) in COVID-19 pandemic findings drawn from China (18, 19), no gender differences were noted in respect to self-isolation or self-quarantine in the present study.

The practical utility of this study is rooted in its potential to guide healthcare planners in making targeted evidenced-based decisions in deploying resources to assist individuals who self-isolate or self-quarantine during the COVID-19 pandemic. The deployment of simple technology like text messaging may be used to provide hopeful and encouraging information to individuals who are isolating or quarantining. The elderly are more likely to self-isolate or self-quarantine and therefore more likely to experience symptoms of stress and anxiety. They may also be less technologically savvy and therefore may not rely much on social media, instead depending on newspapers, radio, and television for entertainment and news. Public health measures should be directed at upskilling the elderly in the use of phones and the internet, as well, news articles on mass media should have a blend of the negative facts about the virus with more positive news about recoveries, and focus less on sensationalism which fuels uncertainty and panic. Guarantees about jobs and income during periods of self-isolation and self-quarantine may help to reduce anxieties about finances and loss of earnings and may deter individuals from disregarding policy guidelines suggesting that they self-isolate or self-quarantine.

Data collection for this study was completed by March 31, 2020, a week into the commencement of public health measures to limit the spread of COVID-19, and thus we are looking at early stage effects. This early stage data capture is a strength of our study as is the inclusion of an anxiety scale. Most studies have investigated post-traumatic stress disorder (PTSD) and depression in the aftermath of disasters and, in that context, generalized anxiety disorder is less frequently studied (20). Anxiety may fluctuate across time (19) during an epidemic, variation that has been attributed to trait anxiety, situation-appropriate coping strategies of avoidance, and personal hygiene practice (23). How the pattern and prevalence of anxiety evolves as the COVID-19 pandemic continues is an ongoing focus of our work.

Limitations of this study include a lack of baseline data on stress, anxiety, and depression levels before self-isolation and self-quarantine measures were implemented in Alberta: our study was initiated shortly after these measures were introduced. Another limitation of the study is that subscribers of the Text4Hope program could be residents who were seeking mental health supports because they were more psychologically impacted by the pandemic than non-subscribers, there-by introducing a selection bias. For example, in a cross-sectional survey of subscribers to Text4Mood (the precursor to the Text4Hope program), about half of those surveyed indicated they signed up for the program to help elevate their mood (51.6 %, *n* = 461) or to help them feel better (49 %, *n* = 440), and a quarter indicated that they signed up for the program to help them worry less (24.5 %, *n* = 219) (10). This suggests that individuals experiencing psychological distress are more likely to enroll on supportive text messaging programs.

Non-response bias may have also affected the results, given the low response rate in our study sample (22). It is possible that non-respondents may differ in a systematic way compared to respondents. For example, they may be more (or less) affected by the pandemic, or may have limitations in literacy or English fluency. Despite the low survey response rate, this study provides useful data about the mental health characteristics of individuals who self-isolate or self-quarantine in the early stages of a pandemic. Our findings present an important initial source of information for government and healthcare planners in determining the nature and quality of services required to address mental health challenges arising during this pandemic, as well as future pandemics that employ self-isolation or self-quarantine measures. Specifically, planning for and implementing virtual care programs including supportive text messages may be a fruitful approach to supporting isolated or quarantined individuals. Our research contributes to a larger program of research that is deliberately responding to the ongoing call for transformation of mental health care service to meet the needs of populations during the COVID-19 pandemic. Our study also aligns with our research program advocacy for mobilizing supportive text message technology (24–32) to reach populations disparately affected by mental health concerns.

## Data Availability Statement

The raw data supporting the conclusions of this article will be made available by the authors, without undue reservation.

## Ethics Statement

The study was reviewed and approved by University of Alberta Human Research Ethics Review Board (Pro00086163). The participants consent was implied if they accessed the online survey and returned completed responses.

## Author Contributions

VIOA conceived and designed the study, including the Text4Hope program, supervised data collection, and performed data analysis. VIOA and NN jointly drafted the manuscript. KM, MH, AG, WV, and SS participated in study design. MH, AG, WV, and SS participated in data collection. AA-A, LU, BC, and AJG critically reviewed the manuscript and contributed to the final draft of the manuscript. All authors reviewed and approved the final draft of the manuscript.

## Conflict of Interest

The authors declare that the research was conducted in the absence of any commercial or financial relationships that could be construed as a potential conflict of interest.

## References

[1] World Health Organization Coronavirus Disease 2019. (2020). Available online at: https://www.who.int/emergencies/diseases/novel-coronavirus-2019 (accessed April 5, 2020).

[2] Coronavirus Disease (COVID-19): Outbreak Update Available online at: https://www.canada.ca/en/public-health/services/diseases/2019-novel-coronavirus-infection.html (accessed April 5, 2020).

[3] Taha S Matheson K Cronin T Anisman H. Intolerance of uncertainty, appraisals, coping and anxiety: the case of the 2009 HINI Pandemic. Br J Health Psychol. (2014) 19:592–605. 10.1111/bjhp.12058 23834735

[4] Brooks SK Webster RK Smith LE Woodland L Wessely S Greenberg N. The psychological impact of quarantine and how to reduce it: rapid review of the evidence. Lancet. (2020) 395:912–20. 10.1016/S0140-6736(20)30460-8 32112714 PMC7158942

[5] Manuell M-E Cukor J. Mother Nature versus human nature: public compliance with evacuation and quarantine. Disasters. (2011) 35:417–42. 10.1111/j.1467-7717.2010.01219.x 21073672

[6] Centers for Disease Control and Prevention Quarantine and Isolation. (2017). Available online at: https://www.cdc.gov/quarantine/index.html (accessed April 5, 2020).

[7] Miles SH. Kaci Hickox. Public Health and the Politics of Fear. Available online at: http://www.bioethics.net/2014/11/kaci-hickox-public-health-and-thepolitics-of-fear/ (accessed April 5, 2020). 10.1080/15265161.2015.1010994 25856593

[8] CMOH Order 02-2020: 2020 COVID-19 Response Available online at: https://open.alberta.ca/dataset/b1efba5f-57ac-44da-a8e0-ceebe6565d72/resource/250393d5-f8a3-409e-9e1a-710e26dbac2d/download/health-cmoh-record-fof-decision-cmoh-02-2020.pdf (accessed April 5, 2020).

[9] Berrouiguet S Baca-García E Brandt S Walter M Courtet P. Fundamentals for future mobile-health (mHealth): a systematic review of mobile phone and web-based text messaging in mental health. J Med Internet Res. (2016) 18:e135. 10.2196/jmir.5066 27287668 PMC4920962

[10] Agyapong VI Mrklas K Juhás M Omeje J Ohinmaa A Dursun SM. Cross-sectional survey evaluating Text4Mood: mobile health program to reduce psychological treatment gap in mental healthcare in Alberta through daily supportive text messages. BMC Psychiatry. (2016) 16:378. 10.1186/s12888-016-1104-2 27821096 PMC5100254

[11] Cohen S Kamarck T Mermelstein R. A global measure of perceived stress. J Health Soc Behav. (1983) 24:385–96. 10.2307/2136404 6668417

[12] Spitzer RL Kroenke K Williams JB Löwe B. A brief measure for assessing generalized anxiety disorder: the GAD-7. Arch Intern Med. (2006) 166:1092–7. 10.1001/archinte.166.10.1092 16717171

[13] Kroenke K Spitzer RL Williams JB. The PHQ-9: validity of a brief depression severity measure. J Gen Intern Med. (2001) 16:606–13. 10.1046/j.1525-1497.2001.016009606.x 11556941 PMC1495268

[14] Wang C Pan R Wan X Tan Y Xu L Ho CS. Immediate psychological responses and associated factors during the initial stage of the 2019 coronavirus disease (COVID-19) epidemic among the general population in China. Int J Environ Res Public Health. (2020) 17:1729. 10.3390/ijerph17051729 32155789 PMC7084952

[15] Nathawad R Roblin PM Pruitt D Arquilla B. Addressing the gaps in preparation for quarantine. Prehosp Disaster Med. (2013) 28:132–8. 10.1017/S1049023X1200180X 23356554 PMC7113027

[16] Jeong H Yim HW Song Y-J Moran K Min J-A Cho J. Mental health status of people isolated due to Middle East respiratory syndrome. Epidemiol Health. (2016) 38:e2016048. 10.4178/epih.e2016048 28196409 PMC5177805

[17] Liu X Kakade M Fuller CJ Fan B Fang Y Kong J. Depression after exposure to stressful events: lessons learned from the severe acute respiratory syndrome epidemic. Compr Psychiatry. (2012) 53:15–23. 10.1016/j.comppsych.2011.02.003 21489421 PMC3176950

[18] Wang Y Di Y Ye J Wei W. Study on the public psychological states and its related factors during the outbreak of coronavirus disease 2019 (COVID-19) in some regions of China. Psychol Health Med. (2020) 30:1–10. 10.1080/13548506.2020.1746817 32223317

[19] Qiu J Shen S Zhao M Wang Z Xie B Xu Y. A nationwide survey of psychological distress among Chinese people in the COVID-19 epidemic: implications and policy recommendations. Gen Psychiatr. (2020) 33:e100213. 10.1136/gpsych-2020-100213 32215365 PMC7061893

[20] Goldmann E Galea S. Mental health consequences of disasters. Annu Rev Public Health. (2014) 35:169–83. 10.1146/annurev-publhealth-032013-182435 24159920

[21] Agyapong VI Hrabok M Juhas M Omeje J Denga E Nwaka B. Prevalence rates and predictors of generalized anxiety disorder symptoms in residents of Fort McMurray six months after a wildfire. Front Psychiatry. (2018) 9:345. 10.3389/fpsyt.2018.00345 30108527 PMC6079280

[22] Fincham JE. Response rates and responsiveness for surveys, standards and the journal. Am J Pharm Educ. (2008) 72:43. 10.5688/aj720243 18483608 PMC2384218

[23] Cheng C Cheung MW. Psychological responses to outbreak of severe acute respiratory syndrome: a prospective, multiple time-point study. J Pers. (2005) 73:261–85. 10.1111/j.1467-6494.2004.00310.x 15660679 PMC7167099

[24] Agyapong VIO Hrabok M Vuong W Gusnowski A Shalaby R Surood S. Implementation and evaluation of a text message-based addiction counseling program (Text4Hope-Addiction Support): protocol for a questionnaire study. JMIR Res Protoc. (2020) 9:e22047. 10.2196/22047 33200993 PMC7709002

[25] Agyapong VIO McLoughlin D Farren CK. Perception of patients with alcohol use disorder and comorbid depression about the usefulness of supportive text messages. Technol Health Care. (2013) 21:31–9. 10.3233/THC-120707 23358057

[26] Agyapong V. Coronavirus Disease 2019 pandemic: health system and community response to a text message (Text4Hope) program supporting mental health in Alberta. Disaster Med Public Health Preparedness. (2020) 14:E5–6. 10.1017/dmp.2020.114 32317038 PMC7198462

[27] O'Reilly H Hagerty A O'Donnell S Farrell A Hartnett D Murphy E. Alcohol use disorder and co-morbid depression: a six-month randomised controlled trial investigating the effectiveness of supportive text messages in aiding recovery. Alcohol Alcohol. (2019) 54:551–8. 10.1093/alcalc/agz060 31361815

[28] Agyapong VIO Juhás M Mrklas K Hrabok M Omeje J Gladue I. Randomized controlled pilot trial of supportive text messages for patients with alcohol use disorder. J Subst Abuse Treat. (2018) 94:74–80. 10.1016/j.jsat.2018.08.014 30243421

[29] Agyapong VIO Juhás M Omeje J Mrklas K Suen V Ohinmaa A. Randomized controlled trial of supportive text messages for patients with depression. BMC Psychiatry. (2017) 17:286. 10.1186/s12888-017-1448-2 28768493 PMC5541655

[30] Agyapong VIO Mrklas K Suen V Rose MS Jahn M Gladue I Supportive text messages to reduce mood symptoms and problem drinking in patients with primary depression or alcohol use disorder: protocol for an implementation research study. JMIR Res Protoc. (2015) 4:e55 10.2196/resprot.4371 25979786 PMC4814207

[31] Agyapong VIO McLoughlin D Farren CK. Six-months outcomes of a randomised trial of supportive text messaging for depression and comorbid alcohol use disorder. J Affect Disord. (2013) 151:100–4. 10.1016/j.jad.2013.05.058 23800443

[32] Agyapong VIO Hrabok M Vuong W Mrklas K Li D Urichuk L Mental health response to the COVID-19 pandemic: effectiveness of a daily supportive text message (Text4Hope) program at six weeks in reducing stress, anxiety, and depression in subscribers. JMIR Ment Health. (2020) 7:e22423 10.2196/22423 33296330 PMC7752184

